# Dosimetric parameter predicting the deterioration of hepatic function after helical tomotherapy in patients with unresectable locally advanced hepatocellular carcinoma

**DOI:** 10.1186/1748-717X-8-11

**Published:** 2013-01-09

**Authors:** Seok Hyun Son, Chul Seung Kay, Jin Ho Song, Sea-Won Lee, Byung Ock Choi, Young Nam Kang, Jeong Won Jang, Seung Kew Yoon, Hong Seok Jang

**Affiliations:** 1 Department of Radiation Oncology, College of Medicine, The Catholic University of Korea, Seoul, Korea; 2 Department of Internal Medicine, College of Medicine, The Catholic University of Korea, Seoul, Korea

**Keywords:** Hepatocellular carcinoma (HCC), Helical tomotherapy, Radiation-induced hepatic toxicity (RIHT), Child-Pugh score (CP score), Dosimetric parameter, The deterioration of hepatic function

## Abstract

**Background:**

The purpose of this study was to identify parameters capable of predicting the deterioration of hepatic function after helical tomotherapy in patients with unresectable locally advanced hepatocellular carcinoma.

**Methods:**

Between March 2006 and February 2012, 72 patients were eligible for this study. All patients received hypofractionated radiotherapy using the TomoTherapy Hi-Art (TomoTherapy, Madison, WI, USA) at Seoul St. Mary's Hospital and Incheon St. Mary's Hospital, the Catholic University of Korea. The radiation dose was a median 50 Gy (range: 40–50 Gy) in 10 fractions to 95% of the planning target volume. Radiation-induced hepatic toxicity was defined as an increase of at least 2 points in the Child-Pugh (CP) score within 3 months after completion of helical tomotherapy.

**Results:**

An increase of at least 2 points in the CP score occurred in 32 of the 72 patients (44.4%). Multivariate logistic regression analysis revealed that pretreatment CP class and V_15Gy_ were significant parameters associated with an increase in CP score (*p* = 0.009 and *p* < 0.001, respectively). The area under receiver operating characteristic curve was 0.863 for V_15Gy_ (*p* < 0.001). For V_15Gy_, with a cutoff value of 43.2%, the accuracy was 0.806 (58/72) with a sensitivity of 0.938 and a specificity of 0.725.

**Conclusions:**

An increase of at least 2 points in the CP score is a radiation dose-limiting factor, and the non-target normal liver receiving a dose more than 15 Gy (V_15Gy_) should be <43.2% to reduce the risk of the deterioration of hepatic function.

## Background

Hepatocellular carcinoma (HCC), which is one of the most common malignant carcinomas worldwide, is a fatal disease that might cause death with severe complication if treated improperly [1,2]. Although a surgical resection is considered the treatment of choice, many patients are either inoperable or the tumor is unresectable at the time of diagnosis. In these cases, transarterial chemoembolization (TACE), percutaneous ethanol injection (PEI), and radiofrequency ablation (RFA) have been used [3-5]. In contrast, radiotherapy (RT) had not been widely used for the treatment of HCC because of the low dose of radiation tolerable for the entire liver, which was not effective for tumor control [6,7]. However, recently reported studies showed that partial volume irradiation of the liver is feasible and a useful tool for the treatment of HCC within an acceptable range of hepatic toxicity [8-11].

In the use of RT for the treatment of HCC, radiation-induced hepatic toxicity (RIHT) is considered a significant dose-limiting factor because of its potential to cause liver failure. This potential underlines the importance of identifying a parameter that can predict, and thus, prevent RIHT. To date, several reports investigating parameters capable of predicting RIHT have been recently published [12-17]; however, these reports are based on clinical data from 3-dimensional conformal radiotherapy (3D-CRT). When compared with 3D-CRT, helical tomotherapy is an advanced technique that can provide a high dose of radiation to the target volume, while reducing the dose to the surrounding normal tissues. But, low to moderate dose of radiation distributed to a much wider region of the normal liver could affect the incidence of RIHT.

Here, we evaluated RIHT reflecting the deterioration of hepatic function in advanced HCC patients treated with helical tomotherapy and identified a parameter capable of predicting RIHT.

## Methods

### Patients

The inclusion criteria for this study were as follows: 1) unresectable locally advanced HCC, 2) prior treatment by hypofractionated helical tomotherapy with a curative aim, 3) a radiation dose of 40–50 Gy in 10 fractions, 4) 2 or more laboratory studies within 3 months after the completion of helical tomotherapy, 5) 1 or more radiologic studies within 3 months after the completion of helical tomotherapy, and 6) no intrahepatic disease progression within 3 months after the completion of helical tomotherapy.

Between March 2006 and February 2012, 72 patients were eligible for this study. All the patients received hypofractionated RT using the TomoTherapy Hi-Art (TomoTherapy, Madison, WI, USA) at Seoul St. Mary's Hospital and Incheon St. Mary's Hospital, the Catholic University of Korea. The patients' clinical and dosimetric data were retrospectively collected following Institutional Review Board approval. The patients' characteristics are shown in Table 1.

**Table 1:** Patient’s characteristics

**Characteristic**	***n***	**(%)**
Gender		
Male	54	75.0
Female	18	25.0
Age (year)		
Median	60	
Range	21-80	
ECOG PS		
0	23	31.9
1	49	68.1
Hepatitis		
No	2	2.8
Yes	70	97.2
HBV	52	72.2
HCV	6	8.3
Others	12	16.7
Liver cirrhosis		
No	16	22.2
Yes	56	77.8
PVTT		
No	30	41.7
Yes	42	58.3
AFP (IU/mL)		
<400	48	66.7
≥400	24	33.3
Child-Pugh class		
A	54	75.0
B	18	25.0
AJCC stage		
II	12	16.7
III	53	73.6
IVA	7	9.7
Previous treatment		
No	7	9.7
Yes	65	90.3
TACE	63	87.5
RFA	7	9.7
PEI	7	9.7
Surgery	9	12.5
Treatment after RT		
No	31	43.1
Yes	41	56.9
TACE	40	55.6
RFA	2	2.8
PEI	2	2.8
Systemic CTx	3	4.2
Radiation dose		
40 Gy/10 fxs	6	8.3
45 Gy/10 fxs	10	13.9
50 Gy/10 fxs	56	77.8

Abbreviations: *ECOG PS* Eastern Cooperative Oncology Group performance status; *HBV* hepatitis B virus; *HCV* hepatitis C virus; *PVTT* portal vein tumor thrombosis; *AFP* alpha-fetoprotein; *AJCC* American Joint Committee on Cancer; *TACE* transcatheter arterial chemoembolization; *RFA* radiofrequency ablation; *PEI* percutaneous ethanol injection; *RT* radiotherapy; *CTx* chemotherapy; *fxs* fractions.

Prior to helical tomotherapy, 65 patients (90.3%) were treated with other locoregional treatments such as TACE, RFA, PEI, and surgery. Within 3 months after the completion of helical tomotherapy, TACE was performed in 41 patients (56.9%) (median: 1 time, average: 1.5 times, range: 1–3 times). In addition, RFA and PEI were performed in 2 patients each (2.8%) and systemic chemotherapy was performed in 3 patients (4.2%).

### Target volume and treatment

The gross tumor volume (GTV) was defined as the tumor volume that was enhanced in the arterial phase and diluted in the delayed phase of computed tomography (CT) scan. The planning target volume (PTV) was generated by adding 5–15 mm to the GTV in 52 of the 72 patients, facilitating asymmetric margin expansion to reduce irradiation to the stomach, duodenum, and small intestine. In the remaining 20 of 72 patients, 4-dimensional CT (4D-CT) was performed to generate the internal target volume to compensate for respiratory-induced liver movement because of the installation of 4D-CT in March 2009 at Seoul St. Mary’s hospital and in March 2011 at Incheon St. Mary’s hospital. Organs at risks such as the total liver, non-target normal liver (NTNL), stomach, duodenum, intestine, kidney, and spinal cord were also contoured for evaluation of the irradiated dose. NTNL volume was the total liver volume minus PTV.

The prescribed radiation dose was a median 50 Gy (range: 40–50 Gy) in 10 fractions to 95% of the PTV. Prior to the actual beam delivery, megavoltage cone-beam CT was performed at the time of every treatment session. Each patient’s set-up and position were corrected with automated image registration, and the anatomical accuracy was always evaluated by a radiation oncologist.

### Evaluation of the radiation-induced hepatic toxicity

RIHT was defined as an increase of at least 2 points in the Child-Pugh (CP) score within 3 months after the completion of helical tomotherapy. CP score, which is calculated on the basis of the serum bilirubin and albumin levels, prothrombin time (PT), and the presence and degree of ascites or encephalopathy, is an assessment of the severity of hepatic function. Thus, an increase in CP score reflects the deterioration of hepatic function [18].

During helical tomotherapy, patients were seen weekly by a physician to evaluate their complaints. After completion of helical tomotherapy, patients were followed-up every 1–2 months. At every visit, physical examinations and blood tests were performed to assess hepatic toxicity. Levels of aspartate transaminase (AST), alanine transaminase (ALT), alkaline phosphatase (ALP), serum albumin, total bilirubin and PT were examined. Ascites and hepatic encephalopathy were also evaluated.

### Parameters for predicting RIHT

The clinical parameters analyzed were gender, age, Eastern Cooperative Oncology Group (ECOG) performance status, pretreatment CP class, American Joint Committee on Cancer (AJCC) stage, pretreatment level of alpha-fetoprotein (AFP) and the presence or absence of hepatitis, liver cirrhosis, portal vein tumor thrombosis (PVTT) and previous treatments.

The dosimetric parameters analyzed were PTV, mean dose of NTNL, the percentage of NTNL volume receiving >5 Gy (V_5Gy_), >10 Gy (V_10Gy_), >15 Gy (V_15Gy_), >20 Gy (V_20Gy_), >25 Gy (V_25Gy_), >30 Gy (V_30Gy_), >35 Gy (V_35Gy_), and >40 Gy (V_40Gy_).

### Statistical analyses

Pearson's chi-square and the independent t-test were used for univariate analysis of the clinical parameters associated with RIHT. Binary logistic regression analysis was used for univariate analysis of dosimetric parameters associated with RIHT. Multivariate analysis was performed using the logistic regression model containing all significant variables according to univariate analysis (selection: stepwise forward). The receiver operating characteristic (ROC) curve was used to estimate the significant dosimetric parameters. All statistical analyses were performed using SPSS ver. 12.0 (SPSS Institute, Chicago, Illinois) and a *p* value of <0.05 was considered significant.

## Results

### Clinical and dosimetric parameters associated with an increase in CP score

An increase of at least 2 points in the CP score occurred in 32 of 72 patients (44.4%) within 3 months after completion of helical tomotherapy. Univariate analysis results showing the associations between clinical parameters and the increased the CP score are summarized in Table 2. Age, gender, pretreatment level of AFP, and the presence of hepatitis and/or liver cirrhosis did not contribute to an increase in CP score after helical tomotherapy. In addition, previous treatments and treatments given after completion of helical tomotherapy did not contribute to an increase in CP score (*p* = 0.374 and 0.394, respectively). In contrast, AJCC stage, the presence of PVTT, and pretreatment CP class were significantly associated with an increase in the CP score after helical tomotherapy (*p* = 0.021, 0.037, and 0.006, respectively).

**Table 2:** Clinical parameters in patients with or without an increase in Child-Pugh score ≥ 2

	**Without (n = 40)**	**With (n = 32)**	***p*value**
Gender			0.584
Male	31	23	
Female	9	9	
Age (years)			0.231
median	62	59	
range	21-80	41-80	
ECOG PS			0.910
0	13	10	
1	27	22	
Hepatitis			0.109
No	0	2	
Yes	40	30	
AFP (IU/mL)			0.094
<400	30	18	
≥400	10	14	
LC			0.228
No	11	5	
Yes	29	27	
PVTT			*0.037
No	21	9	
Yes	19	23	
CP class			*0.006
A	35	19	
B	5	13	
AJCC stage			*0.021
II	11	1	
III	26	27	
IVA	3	4	
Previous treatment			0.374
No	5	2	
Yes	35	30	
Treatment after RT			0.394
No	19	12	
Yes	21	20	

Abbreviations: *ECOG PS* Eastern Cooperative Oncology Group performance status; *AFP* alpha-fetoprotein; *LC* liver cirrhosis; *PVTT* portal vein tumor thrombosis; *CP class* Child-Pugh class; *AJCC* American Joint Committee on Cancer; *RT* radiotherapy.

*Significant parameters in univariate analysis.

Univariate analysis of the associations between the dosimetric parameters and the increase in CP score showed that PTV, mean dose of NTNL, V_5Gy_, V_10Gy_, V_15Gy_, V_20Gy_, V_25Gy_, V_30Gy_ and V_35Gy_ were significantly associated with an increase in the CP score (Table 3).

**Table 3:** Dosimetric parameters in patients with or without an increase in Child-Pugh score ≥ 2

**Parameters**	**Without (n = 40)**	**With (n = 32)**	***p*value (95%CI)**	**ROC curve**	
				**AUC**	***p*value**
PTV (cm^3^)	103.8 ± 102.8	227.1 ± 193.5	0.004 (1.002-1.011)	0.741	<0.001
Mean dose of NTNL (Gy)	14.9 ± 4.1	20.2 ± 3.2	<0.001 (1.209-1.708)	0.839	<0.001
V_5Gy_	76.6 ± 17.5	91.2 ± 7.6	0.001 (1.040-1.153)	0.765	<0.001
V_10Gy_	57.1 ± 19.1	79.2 ± 10.7	<0.001 (1.048-1.140)	0.830	<0.001
*V_15Gy_	39.2 ± 14.8	61.3 ± 11.9	<0.001 (1.064-1.180)	0.863	<0.001
V_20Gy_	27.1 ± 11.0	44.0 ± 11.8	<0.001 (1.069-1.199)	0.852	<0.001
V_25Gy_	19.4 ± 8.6	30.5 ± 10.1	<0.001 (1.062-1.204)	0.804	<0.001
V_30Gy_	14.8 ± 7.6	21.6 ± 8.3	0.002 (1.040-1.187)	0.738	0.001
V_35Gy_	10.5 ± 6.2	15.4 ± 7.1	0.006 (1.033-1.205)	0.706	0.003
V_40Gy_	7.2 ± 5.1	9.9 ± 5.1	0.053 (0.999-1.190)	-	-

Abbreviations: *CI* confidence interval; *ROC* receiver operating characteristic; *AUC* area under curve; *PTV* planning target volume; *NTNL* non-target normal liver.

*Significant parameter on multivariate logistic regression analysis.

**Figure 1:**
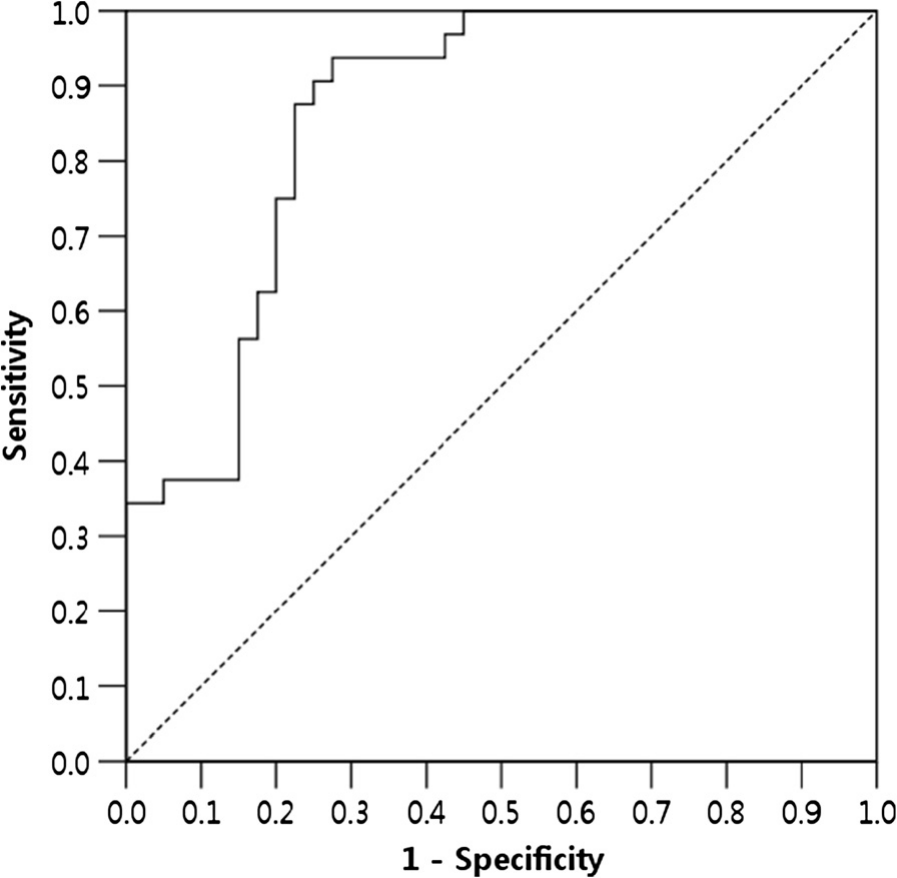
**Receiver operating characteristic curve for V**_**15Gy**_**.**

Multivariate logistic regression analysis confirmed that pretreatment CP class and V_15Gy_ were significant parameters associated with an increase in the CP score (*p* = 0.009 and *p* < 0.001, respectively). As shown in Figure 1, the area under the ROC curve (AUC) was 0.863 for V_15Gy_ (*p* < 0.001), indicating that V_15Gy_ with a cutoff value of 43.2% was an appropriate value to predict an increase in the CP score. An increase in the CP score was observed in 2 of 30 patients (6.7%) with a V_15Gy_ of ≤43.2% and in 30 of 43 patients (69.8%) with a V_15Gy_ of >43.2%. For V_15Gy_, with a cutoff value of 43.2%, the accuracy was 0.806 (58/72) with a sensitivity of 0.938 and a specificity of 0.725.

### Normal liver dose-volume histogram (DVH) reflecting the risk of the deterioration of hepatic function

**Figure 2:**
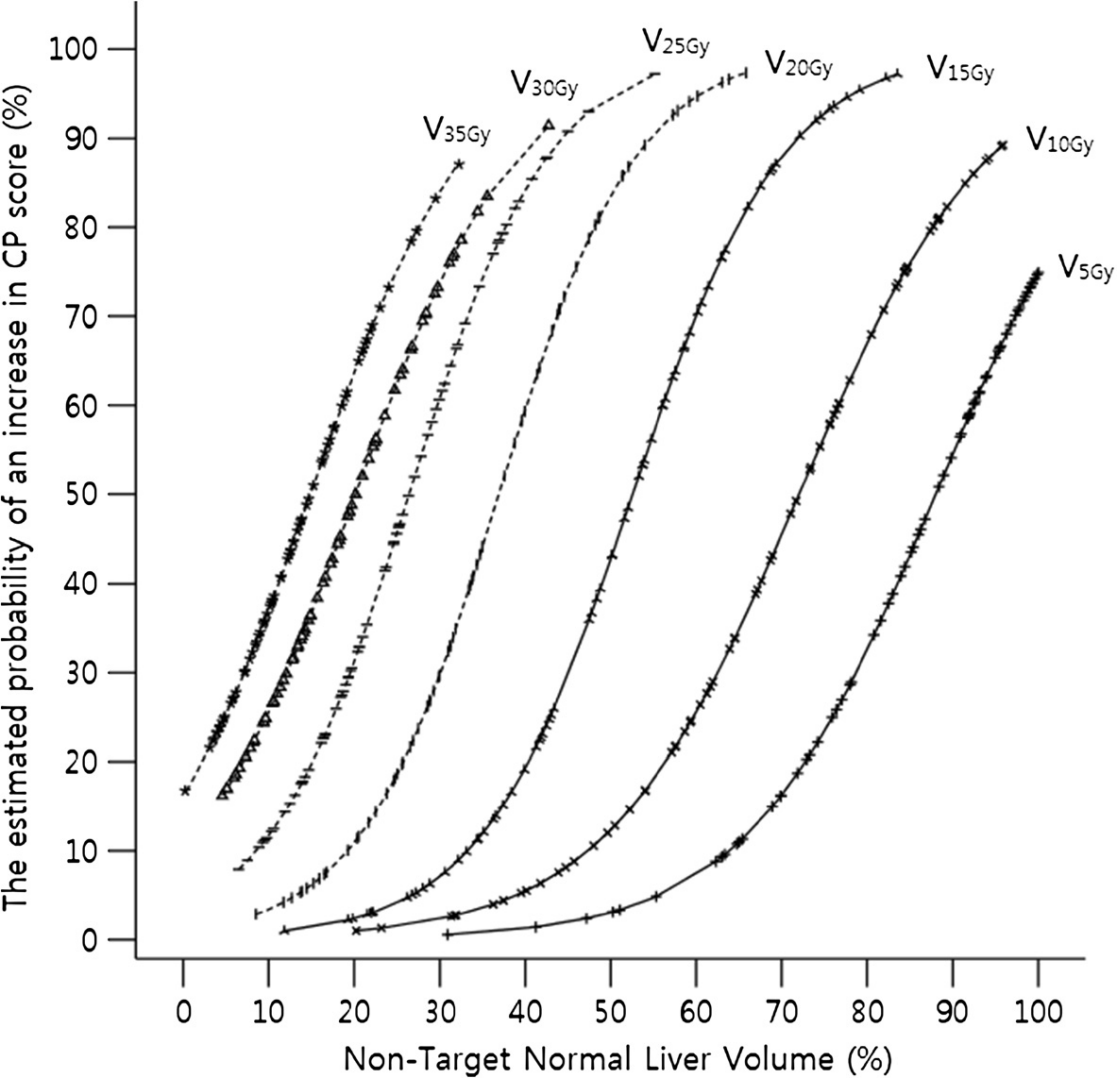
**The estimated probability of an increase in CP score for V**_**5Gy, **_**V**_**10Gy, **_**V**_**15Gy, **_**V**_**20Gy, **_**V**_**25Gy, **_**V**_**30Gy, **_**and V**_**35Gy**_**.**

**Figure 3:**
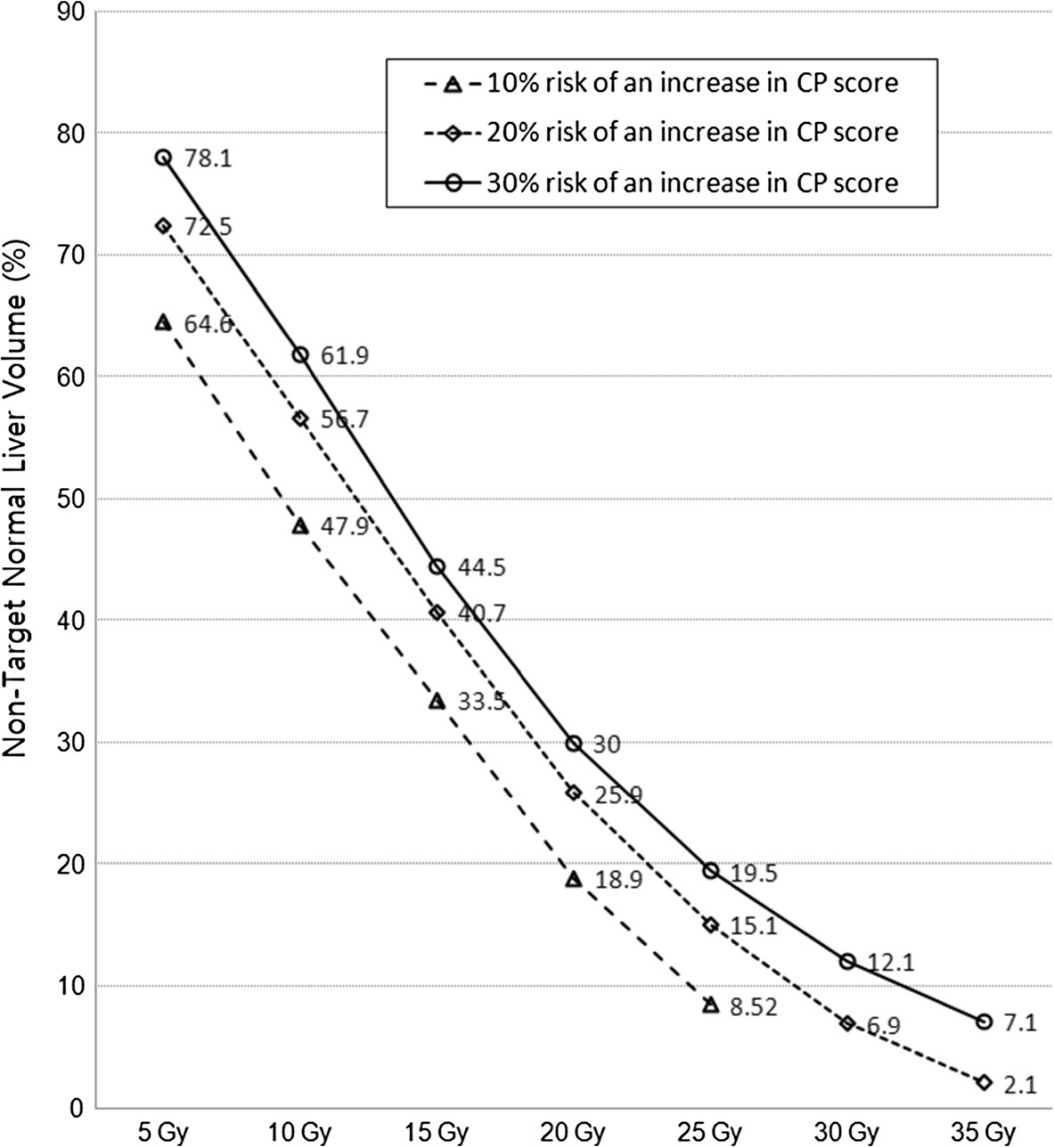
**The normal liver dose-volume histogram indicating a 10**%**, 20**%**, and 30**% **risk of an increase in CP score.**

Close correlations were demonstrated between dosimetric parameters that were shown to be significant in the univariate analysis (V_5Gy_ vs. V_10Gy_, *r* = 0.890, *p* < 0.001; V_10Gy_ vs. V_15Gy_, *r* = 0.936, *p* < 0.001; V1_5Gy_ vs. V_20Gy_, *r* = 0.945, *p* < 0.001; V_20Gy_ vs. V_25Gy_, *r* = 0.962, *p* < 0.001; V_25Gy_ vs. V_30Gy_, *r* = 0.916, *p* < 0.001; V_30Gy_ vs. V_35Gy_, *r* = 0.904, *p* < 0.001). Although V_15Gy_ was the only significant dosimetric parameter found in multivariate analysis, other parameters (V_5Gy_, V_10Gy_, V_20Gy_, V_25Gy_, V_30Gy_ and V_35Gy_) that were shown to be statistically significant in univariate analysis also demonstrated similar relationship patterns in their estimated probability curves (Figure 2). Additionally, the area under the ROC curve of each of these parameters was regarded as a good value with statistical significance (Table 3). From these curves, the values indicating a 10%, 20% and 30% risk of an increase in the CP score were obtained. These values correspond to the normal liver DVH reflecting the risk of the deterioration of hepatic function. It could provide a treatment planning guideline to reduce the risk of developing the deterioration of hepatic function (Figure 3).

## Discussion

Ablation, chemoembolization, and RT have been used for the treatment of unresectable locally advanced HCC. Because RT alone cannot achieve a complete response in most cases, other locoregional treatments, such as TACE, RFA, and PEI, have been used repeatedly prior to or after RT. After completion of RT, transient elevation of hepatic enzymes such as AST, ALT, and ALP commonly occurs, but, in many cases, these kinds of hepatic toxicities are recovered in a few months [14]. However, if hepatic function deteriorates because of radiation, other necessary treatments cannot be performed in a timely manner. Since no specific treatment for this condition exists except conservative care, it is also important to reduce the development of the deterioration of hepatic function. Thus, it is important to identify a parameter that can predict the deterioration of hepatic function and to develop a plan for RT using the parameter and its values.

The relationship between the radiation dose to liver volume and the incidence of hepatic toxicities has been studied previously [12-15,17]. Radiation-induced liver disease (RILD) is a traditionally accepted concept of hepatic toxicity [7]. In the past, classic RILD was a serious manifestation of hepatic toxicity caused by irradiation of 30–35 Gy to the entire liver. However, the incidence of classic RILD has been lowered, since partial volume irradiation has become more common. Other authors have reported the parameters predicting the non-classic RILD or the elevation of hepatic enzymes ≥ grade 2 or 3 according to Radiation Therapy Oncology Group toxicity criteria or Common Terminology Criteria for Adverse Events (CTCAE) and their cut-off values to present a guideline for radiation planning [12,14,15,17].

Our study differs from previous studies in 3 aspects. First, the presence or absence of an increase of at least 2 points in the CP score as an end-point of RIHT was used for analysis. A few authors have analyzed the elevation of hepatic enzymes (AST, ALT and ALP) as end points to find significant parameters that predict hepatic toxicities [12-15,17]. Kim *et al.* showed that the elevation of hepatic enzymes was transient and recovered within a median of 2 months after the completion of RT [14]. However, Furuse *et al.* showed that hypoalbuminemia, hyperbilirubinemia and ascites were important adverse hepatic events that occur after the application of RT to treat advanced HCC, and these events seriously affected survival [19]. An albumin, bilirubin and ascites were used to calculate the CP score. In our previous study, the progression of CP class was analyzed as a useful radiation dose-limiting factor predicting the deterioration of hepatic function, whereas the elevation of hepatic enzymes according to the CTCAE scale was inappropriate as a useful end point [20]. Liaw *et al.* also used an increase of at least 2 points in the CP score to evaluate the deterioration of hepatic function in patients who were treated with lamivudine [18]. Therefore, the CP score is appropriate for the assessment of hepatic function, and an increase of at least 2 points in the CP score should be considered the dose-limiting factor.

Another difference between our study and previous studies is that only patients treated with helical tomotherapy were included in the present study. Previous studies regarding the dosimetric parameters predicting hepatic toxicity were based on the data from 3D-CRT. The planning and delivery method of helical tomotherapy is different from that of 3D-CRT. Because helical tomotherapy is delivered continuously from all angles around the patient via a ring gantry, in which the linear accelerator is mounted, a low to moderate radiation dose is delivered to a much wider region of liver. Because of this characteristic, the parameter and its cut-off value could be different between 3D-CRT and helical tomotherapy. According to Kim *et al.*, V_30_ was demonstrated as a significant parameter in patients treated with conventional fractionated RT [14], and according to Liang *et al.*, V_20_ was a significant parameter in patients treated with hypofractionated RT (4–6 Gy per fraction) [16]. In our study, a significant parameter is V_15Gy_, which is a parameter that corresponds to lower doses than those of above studies. The cut-off value of 43.2% for V_15Gy_ in our study is lower than that in the above studies (60% for V_30_ in the study of Kim *et al*. and 48.5% for V_20_ in the study of Ling *et al*.). This result is probably because of the characteristic planning and delivery method of helical tomotherapy and indicates that, to reduce the risk of the deterioration of hepatic function, a wider region of normal liver should be preserved from a low to moderate dose of radiation.

The third differences between our study and previous studies is that V_15Gy_ was confirmed the only significant dosimetric parameter in multivariate analysis. However, because of the significant correlations between dosimetric parameters shown in univariate analysis, consideration of the values of these parameters could be helpful in the treatment planning phase. Based on the estimated probability curves of V_5Gy_, V_10Gy_, V_15Gy_, V_20Gy_, V_25Gy_, V_30Gy_, and V_35Gy_, which were statistically significant in univariate logistic and ROC curve analyses, we presented the normal liver DVH indicating a 10%, 20%, and 30% risk of the deterioration of hepatic function (Figure 3). This curve could be used as a reference tolerance curve to evaluate treatment plans.

In conclusion, an increase of at least 2 points in the CP score is a radiation dose-limiting factor, and the non-target normal liver receiving a dose more than 15 Gy (V_15Gy_) should be <43.2% to reduce the risk of the deterioration of hepatic function. Moreover, the proposed normal liver DVH could be useful as a reference curve to evaluate the dose to the liver.

## Abbreviations

CP score: Child-Pugh score; HCC: Hepatocellular carcinoma; RIHT: Radiation-induced hepatic toxicity; TACE: Transarterial chemoembolization; PEI: Percutaneous ethanol injection; RFA: Radiofrequency ablation; RT: Radiotherapy; 3D-CRT: 3-dimensional conformal radiotherapy; GTV: Gross tumor volume; PTV: Planning target volume; NTNL: Non-target normal liver; PT: Prothrombin time; AST: Aspartate transaminase; ALT: Alanine transaminase; ALP: Alkaline phosphatase; ECOG: Eastern Cooperative Oncology Group; AJCC: American Joint Committee on Cancer; AFP: Alpha-fetoprotein; PVTT: Portal vein tumor thrombosis; ROC: Receiver operating characteristic; DVH: Dose-volume histogram; RILD: Radiation-induced liver disease; CTCAE: Common Terminology Criteria for Adverse Events; LC: Liver cirrhosis; CTx: Chemotherapy; fxs: Fractions; CI: Confidence interval; AUC: Area under curve.

## Competing interests

The authors declare that they have no competing interests.

## Authors’ contributions

SHS, JHS, SWL, BOC and HSJ collected clinical data and interpreted the results. SHS and YNK collected and evaluated dosimetric data. SHS, CSK, HSJ, JWJ, and SKY took care of patients. SHS, CSK, JWJ, SKY and HSJ were involved in study design. SHS performed statistical analysis and drafted the manuscript. All the authors have read and approved the final draft.
